# Factors associated with job satisfaction among graduate nursing faculties in Nepal

**DOI:** 10.1186/s12912-019-0379-2

**Published:** 2019-11-28

**Authors:** Abja Sapkota, Usha K. Poudel, Jyotsana Pokharel, Pratima Ghimire, Arun Sedhain, Gandhi R. Bhattarai, Binu Thapa, Tulza K.C

**Affiliations:** 1 Department of Nursing, Nepal Medical College, Gokarneshwor- 44602, Attarkhel, Jorpati Kathmandu, Nepal; 2 Pokhara Nursing Campus, Pokhara, Nepal; 3 Nepal Medical College, Attarkhel, Jorpati, Kathmandu, Nepal; 4 Chitwan Medical College, Bharatpur, Chitwan Nepal; 5 OptumInsight, Minneapolis MN, USA; 6 Kathmandu University School of Medical Sciences, Dhulikhel, Kavre Nepal; 7 Maharajgunj Nursing Campus, Maharjgunj, Kathmandu, Nepal

**Keywords:** Nursing faculty, Job satisfaction, Organizational characteristics

## Abstract

**Background:**

Job satisfaction among nursing faculty is critical to improving quality of nursing education, producing future nurses who will contribute directly to the health of patients at a local and national level. This study explores factors associated with job satisfaction among graduate nursing faculties in different universities of Nepal.

**Methods:**

A cross-sectional study was conducted among nursing faculty with at least one year of teaching in their respective institutions. A 36-items job satisfaction questionnaire with 6-point Likert type responses was administered online. The questionnaire was pre-tested with 30 faculties pooled from multiple institutions. Link to the final survey was sent via e-mail to 327 nursing faculties working in 39 nursing colleges. Respondents were contacted by phone as a follow up to the email to politely remind them about the survey. Data analysis was carried out with SAS University Edition software. Chi-Square test and t-test were used for simple descriptive analysis. A multivariate binary logistic regression model was used to identify the significant factors associated with nursing faculties’ job satisfaction. Adjusted odds ratio was calculated and significance was considered at *p* ≤ 0.05 with 95% confidence interval.

**Results:**

The response rate was 54.4%. After retrospective cleaning of data, usable response rate was 52.3% (*n* = 171). The average age of the nursing faculties was 36.8 ± 7.0 years. Based on the overall job satisfaction score, 36.8% nursing faculties were satisfied with their current job. The coefficient for Cronbach’s alpha was 0.895 suggesting very good reliability of the overall measure. The significant factors associated with job satisfaction were the involvement of the faculties in decision making process related to the department (OR = 4.83) and adequate access to reference materials (OR = 2.90).

**Conclusions:**

This study suggests that nursing faculties have positive attitude towards their job but are dissatisfied with the benefits offered to them and the operating condition of their institutions. Expanding the teaching learning resources, such as reference books, subscription to journals, and continuing education opportunities for nursing faculties through participation in professional meetings would be helpful in improving the quality of nursing education in Nepal.

## Background

Job satisfaction is an important component of improving job performance and maintaining the overall quality of work in any organization. Job satisfaction has been defined as the fulfilment of an employee’s expectations for the work he or she performs [1]. It has also been described as a person’s attitude with a correlation between expectations and outcomes at work [2]. It is well established that persons who are satisfied with their job tend to be more creative and innovative for better organizational performance [3]. Therefore, the result of job satisfaction will have an impact not only at the individual level but also in the institutional, societal, and national level. Multiple factors affect a person’s job satisfaction, including pay, benefits and promotions, working condition, leadership and social relationship, diversities of tasks involved, and opportunities and challenges [4, 5].

Nursing teachers are expected to perform multiple tasks that range from teaching nursing students, undertaking research activities, fulfilling an administrative role, facilitating support of staff in practice and providing patient care [6]. In nursing education, it is quite important to maintain and evaluate the satisfaction of teaching faculties because they are related to the production of nursing personnel who provide firsthand health service to the patients. The production of quality nurses with better values depends, to some extent, on the quality of a teacher [7]. When nursing faculties are satisfied with their job, they are more passionate about their work, delivering a higher quality education. Higher job satisfaction would result in lower faculty attrition, increased reputation of the institution, and higher recruitment of most qualified students.

Job satisfaction is a prime global concern and an important facet of any job, yet it has been rarely discussed and explored, especially in developing countries like Nepal. Although limited studies have explored few components of job satisfaction [8, 9], there is paucity of information on the overall job satisfaction of nursing faculties at the national level. It is imperative to understand their needs and expectations and address them sooner than later. This study aims to explore the level of job satisfaction and factors associated with it among the nursing faculties working in seven different Universities and Health Institutes in Nepal.

## Methods

### Study population

A cross-sectional study was conducted to explore the level of job satisfaction and factors associated with it among graduate nursing faculties in Nepal. At the time of the study initiation, 54 nursing colleges affiliated to seven universities offered baccalaureate or masters’ level nursing degrees. All the faculty members who held at least a master’s degree in nursing with current teaching responsibility at bachelors’ level or higher for more than one year at the same institution were considered the population for the study. A census sampling technique was applied for the study. The name of the college was identified through a web search. Among the 54 colleges, four colleges could not be reached due to scheduling conflict, seven did not consent to participate in the study, and four colleges did not have faculties with a master’s degree. The number of nursing faculties with at least a masters’ degree from the remaining 39 colleges was 357.

### Ethical clearance

As the participants were from different institutions, ethical clearance was obtained from the institutional review committees of both Nepal Medical College (NMC), and Nepal Health Research Council (NHRC) before the initiation of the study. Upon approval from IRB, the campus chief from each nursing college was sent a formal letter to receive their institutional approval to conduct the study with their faculties. The respondents were asked to read the instructions and given an opportunity to decide whether would accept or decline the survey.

### Instrument

The instrument was divided into three sections – the first and second sections included sociodemographic and organization related information. These sections were constructed by the researchers through literature search [10] and discussion with the experts. Third section consisted of a 36-item ‘job satisfaction survey’ developed by Spector [11] and used with his permission. Each question included 6-point Likert scale responses (1: disagree very much, 2: disagree moderately, 3: disagree slightly, 4: agree slightly, 5: agree moderately, and 6: agree very much). These questions covered nine different domains and included four questions in each domain. The total score ranged from 108 to 216 and domain scores ranged from 4 to 24.

The questionnaire was posted online with the help of an expert in information technology. A link to the instrument was emailed to 30 randomly selected faculties as a pretest. They were followed up via email on a weekly basis for one month. A valid response was received from 56.6% of the faculties during pretesting. Data from these faculties were excluded from the main study.

### Data collection

The final survey was sent to 327 faculties via a link to the online questionnaire (after excluding 30 pre-test sample). At least one facilitator was identified from each college, who would coordinate the timely delivery of responses from nursing faculties. These facilitators explained the research to participants and collected the mailing addresses of the nursing faculties, which were then sent to researchers. The researchers prepared a list of the faculties and then sent the questionnaire by mail. The researchers remained in frequent contact with the facilitators throughout the data collection period. The facilitators were provided with a remuneration of Nepalese rupees 500 (equivalent to USD 5) for coordinating the research at local level.

**Fig. 1:**
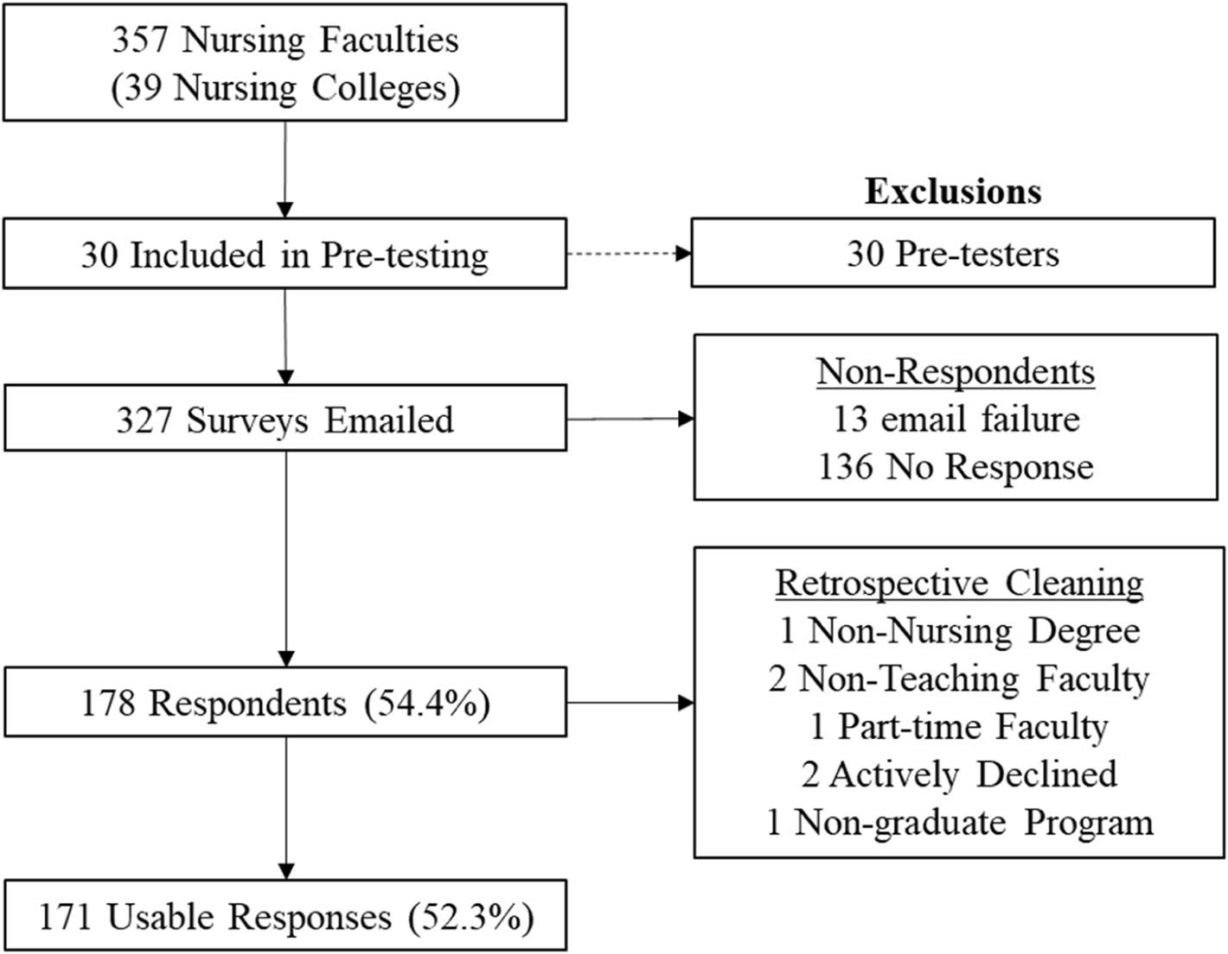
Recruitment process of nursing faculties

Responses were received from 178 (54.4%) of the population. A retrospective cleaning was done to remove those with less than one year of service, those submitted a ‘declined’ response, or those currently not teaching at a graduate (bachelor and above) program. After this cleaning, 171 usable responses (52.3%) were included in the final analysis (Fig. 1).

### Data analysis

The data analysis for this paper was done using SAS/STAT software, Version 14. Copyright© 2017 SAS Institute Inc. SAS and all other SAS Institute Inc. product or service names are registered trademark or trademarks of SAS Institute Inc., Cary, NC, USA. Simple descriptive analysis was done for data exploration. Chi-square test of dependence (for categorical variables) and t-tests (for continuous variables) were performed to check the relationship between job satisfaction and individual variables. Multivariate binary logistic regression model was used to identify factors associated with nursing faculties’ job satisfaction. Adjusted odds ratio was calculated and significance was considered at *p* ≤ 0.05 at CI 95%.

Due to the design constrain during the delivery of online questions, a few demographics and institutional-related questions had some missing values. The missing rate was never higher than 7.5%. Simple imputation was done by replacing the missing values by the mean (continuous variables such as age) or largest class (such as involvement in departmental decision making). There were no missing values in the response for job satisfaction questions.

## Results

### Research participants’ profile

Out of 327 nursing faculties who were sent the questionnaire, 171 (52.3%) were included in the final analysis. The average age was 36.8 ± 7.0 years (range 26 to 57 years). Only 2.9% were male faculties. Most of the faculty had Master of Nursing (MN) degree (55.6%), followed by Master of Science in Nursing (MSN, 40.9%) and Doctorate in Nursing (PhD, 3.5%).

Among the total respondents (*n* = 171), 60.8% were from private colleges and 39.2 were from public colleges. Overall, 58.5% faculties were permanent (tenured), however there were disparities in tenure status among private colleges (45.2%) and public colleges (79.1%). Exactly two thirds of the faculties (66.7%) worked as a Lecturer or lower position, followed by 18.7% as Associate Professor or higher level, and 14.6% as Assistant Professor. 38.0% of faculty had specialization in adult health (adult health nursing, medical-surgical nursing, critical care nursing and general nursing), followed by women’s health (maternal health and women’s health; 26.9%), and child health (pediatrics and children’s health; 17.5%). Only 10.5 and 7.0% were specialized in psychiatric and mental health and community health, respectively.

### Job satisfaction score

Slightly over one third (36.8%) of the graduate nursing faculties were satisfied with their job. Dissatisfaction about their current job was observed only in 14.6% faculties, and a majority (48.5%) had ambivalent feeling towards their job. Among the nine job satisfaction domains, highest job satisfaction was observed in coworkers (81.3%), followed by the nature of job (71.3%), communication (70.8%), and supervision (63.2%). Most dissatisfaction was towards lack of promotion (56.1%), contingency rewards (44.4%), operating condition (44.4%), pay (40.9%), and fringe benefits (35.1%). Only two domains, pay and supervision, have acceptable reliability score (Cronbach’s alpha > = 0.70). Hence, individual domain scores are not analyzed further, and the satisfaction categories are presented for informational purpose. (Table 1).

**Table 1:** Job Satisfaction of graduate nursing faculties

Job Satisfaction Domains	Score (n = 171)	Satisfied^1^		Ambivalent		Dissatisfied		Reliability^2^
	μ ± σ	Count	%	Count	%	Count	%	
Pay	13.8 ± 4.9	59	34.5%	42	24.6%	70	40.9%	0.771
Promotion	12.0 ± 4.1	42	24.6%	33	19.3%	96	56.1%	0.446
Supervision	16.7 ± 4.5	108	63.2%	39	22.8%	24	14.0%	0.795
Fringe Benefits	14.1 ± 4.3	62	36.3%	49	28.7%	60	35.1%	0.527
Contingency Rewards	13.1 ± 4.1	50	29.2%	45	26.3%	76	44.4%	0.569
Operating Conditions	12.9 ± 3.2	30	17.5%	65	38.0%	76	44.4%	0.098
Coworkers	18.3 ± 3.4	139	81.3%	25	14.6%	7	4.1%	0.675
Nature of work	17.1 ± 3.4	122	71.3%	30	17.5%	19	11.1%	0.601
Communication	17.3 ± 3.8	121	70.8%	31	18.1%	19	11.1%	0.606
Overall	135.3 ± 24.8	63	36.8%	83	48.5%	25	14.6%	0.895

Notes

^1^Overall satisfaction - Satisfied: > = 144, Ambivalent: 109–143, Dissatisfied: 36–108

^1^Sub-domain satisfaction - Satisfied: > = 16, Ambivalent: 13–15, Dissatisfied: 4–12

^2^Reliability or internal construct validity is given as Cronbach’s alpha coefficient

The primary objective of this study was to explore factors associated with overall job satisfaction among the graduate nursing faculty. Since majority of the respondents were ambivalent with few respondents expressively dissatisfied, we grouped these two categories as not satisfied as opposed to the respondents who were satisfied. This would allow us to run a multiple logistic regression model with a binary dependent variable (satisfied vs not satisfied).

### Sociodemographic characteristics of graduate nursing faculties by job satisfaction

The details of sociodemographic and organizational characteristics of the faculties are shown in Table 2.

**Table 2:** Sociodemographic characteristics of graduate nursing faculty

Characteristics	Total (*N* = 171)		Not-Satisfied (n0 = 108)	Satisfied (n1 = 63)	*p*-value^1^	Effect Size^2^
	Count	% or μ ± σ	% or μ ± σ	% or μ ± σ		
Age category						
35 or younger	91	53.2%	61.1%	39.7%	0.011	0.230
36 to 45 years	56	32.7%	29.6%	28.1%		
46 and older	24	14.0%	9.3%	22.2%		
Sex						
Female	166	97.1%	99.1%	93.7%	0.056	0.155
Male	5	2.9%	0.9%	6.4%		
Current Position						
Lecturer or lower	114	66.7%	74.1%	54.0%	0.027	0.206
Assistant Professor	25	14.6%	11.1%	20.6%		
Associate Professor +	32	18.7%	14.8%	25.4%		
Highest Degree						
Masters in Nursing	95	55.6%	56.5%	54.0%	0.781	0.054
MSc in Nursing	70	40.9%	40.7%	41.3%		
PhD in Nursing	6	3.5%	2.8%	4.8%		
Specialty in Nursing						
Child Health	30	17.5%	16.7%	19.1%	0.785	0.101
Community Health	12	7.0%	7.4%	6.4%		
Adult Health	65	38.0%	40.7%	33.3%		
Mental Health	18	10.5%	11.1%	9.5%		
Maternal Health	46	26.9%	24.1%	31.8%		
Primary Responsibility						
BSN Level	146	85.4%	90.7%	76.2%	0.009	0.199
MSN Level	25	14.6%	9.3%	23.8%		
Tenure status						
Temporary	71	41.5%	51.9%	23.8%	0.000	0.275
Permanent	100	58.5%	48.1%	76.2%		
Teaching Experience (Years)						
After Master’s degree		5.5 ± 4.8	4.7 ± 3.9	6.8 ± 5.7	0.014	0.209
At current institution		4.9 ± 4.6	3.7 ± 3.0	7.1 ± 6.0	<.0001	0.362
At current position		3.2 ± 3.1	2.7 ± 2.0	3.9 ± 4.4	0.054	0.176
^3^Salary and Benefits (‘00,000)						
Monthly Gross Salary (NRs)		5.5 ± 1.5	5.2 ± 1.1	6.1 ± 2.0	0.001	0.307
Monthly Basic Salary (NRs)		3.6 ± 1.5	3.3 ± 1.0	4.3 ± 1.9	0.000	0.327
Age (in years)		36.8 ± 7.0	35.7 ± 6.2	38.8 ± 7.8	0.008	0.217

Notes

^1^pvalue: *p*-values are based on chi-square test statistics for categorical variables and t-test statistics for continuous variables between satisfied and not-satisfied

^2^Effect size: the magnitude and direction of relationship with outcome are based on Cramer’s V statistics for categorical variables and point biserial correlation (Pearson) for continuous variables

^3^Salary levels: basic salary is the fixed monthly salary whereas gross salary includes provident fund, grades, special allowances

### Organizations related characteristics of nursing faculties by job satisfaction

Majority of the nursing faculties (60.8%) were from private organizations and 39.2% from the public institutions. Public institutions usually offer provident fund, grade, and promotional opportunities for their employees. In recent years, private institutions are also catching up in providing these benefits to both their tenured and non-tenured employees. For example, 76.6% faculties responded positively about grade opportunity and 63.7% reported having provident fund benefits even though the overall employment in public colleges was 39.2% and tenure (permanent) rate was only 58.5%.

A grade in Nepalese context is a periodic increment in the basic salary of employees after they have completed certain years of service. In other word, it can be defined as steps within the same grade level in the USA. On the other hand, a provident fund is a pension fund scheme for employees of both the public and private sectors in Nepal. This fund is managed by a government institution which invests the money to generate profit for the depositors. Under this scheme, eligible employees contribute 10% of their basic salary and employer matches the fund equally. This scheme operates similar to a 401(K) savings plan in the USA.

Healthcare benefits were reported by 74.3% of the respondents. Healthcare benefits in Nepalese context is an employer-provided welfare scheme that provides limited medical care within their network for an employee and their dependents. Immediate family members including spouse and children plus parents of both spouses are usually covered by this benefit.

A majority of respondents reported the availability and easy access to textbooks (74.9%), reference books (66.1%), nursing and medical journals (57.3%) and internet facilities (83.6%). **(**Table 3**).**

**Table 3:** Organization related characteristic of nursing faculty

Characteristics	Total		Not-Satisfied	Satisfied	p-value^1^	Effect Size^2^
	(N = 171)		(n_0_ = 108)	(n_1_ = 63)		
	Count	%	%	%		
Type of Institution						
Private	104	60.8%	67.6%	49.2%	0.018	0.182
Public	67	39.2%	32.4%	50.8%		
Promotional opportunity	97	56.7%	47.2%	73.0%	0.001	0.251
Grade opportunity	131	76.6%	69.4%	88.9%	0.004	0.222
Provident fund benefits	109	63.7%	50.0%	87.3%	<.0001	0.374
Healthcare benefits	127	74.3%	66.7%	87.3%	0.003	0.228
Involved in decision making						
Never or Rarely	55	32.2%	40.7%	17.5%	0.002	0.274
Sometimes	59	34.5%	34.3%	34.9%		
Often or Always	57	33.3%	25.0%	47.6%		
Provision of supervisory Pay	56	32.7%	33.3%	31.8%	0.831	−0.016
Provision of clinical Pay	31	18.1%	14.8%	23.8%	0.141	0.113
PM clinical	147	86.0%	91.7%	76.2%	0.005	−0.215
AM clinical starting ≥ 8 am	79	46.2%	41.7%	54.0%	0.120	0.119
Clinical duty ≥ 7 h	77	45.0%	39.8%	54.0%	0.073	0.137
Overall workload						
Normal (≤ 41 h)	94	55.0%	59.3%	47.6%	0.140	0.113
Overload (≥ 42 h)	77	45.0%	40.7%	52.4%		
Teaching load per year						
≤ 124 h	75	43.9%	38.0%	54.0%	0.042	−0.156
≥ 125 h	96	56.1%	62.0%	46.0%		
Professional Development						
≤ 4 days	95	55.6%	65.7%	38.1%	0.000	0.268
≥ 5 days	76	44.4%	34.3%	61.9%		
Adequate textbook	128	74.9%	66.7%	88.9%	0.001	0.247
Adequate reference book	113	66.1%	55.6%	84.1%	0.000	0.291
Adequate Med/Nursing Journals	98	57.3%	45.4%	77.8%	<.0001	0.316
Adequate internet facilities	143	83.6%	77.8%	93.7%	0.007	0.207
Question setting for tests	85	49.7%	41.7%	63.5%	0.006	0.211
Benefits offered (μ = Rs56K)						
Below average	118	69.0%	75.9%	57.1%	0.010	0.196
Above average	53	31.0%	24.1%	42.9%		

Notes

^1^*p*-value: all p-values are based on Chi-square test statistics

^2^Effect size: the magnitude and direction of relationship of all categorical variables with the outcome are based on Cramer’s V test statistics

### Characteristics associated with job satisfaction

The descriptive results showed that some of the variables have a stronger association with job satisfaction than others. In order to evaluate the effect of each of these factors while simultaneously controlling for other factors, a multiple logistic regression model was used. All variables with an effect size greater than 0.15 (see the correlation coefficient or Cramer’s V statistics in Tables 2 and 3 above) were included in the initial model. Univariate logistic regression (not shown in these tables) also suggested the selection of these variables. All variables that were selected based on the high correlation with job satisfaction were further evaluated for possible collinearity and strong association among each other. Cramer’s V test statistics were used to check for each pair of categorical variables. A cutoff point of 0.50 was established for unusually high correlation at which point two variables were probably measuring the same concept.

**Fig. 2:**
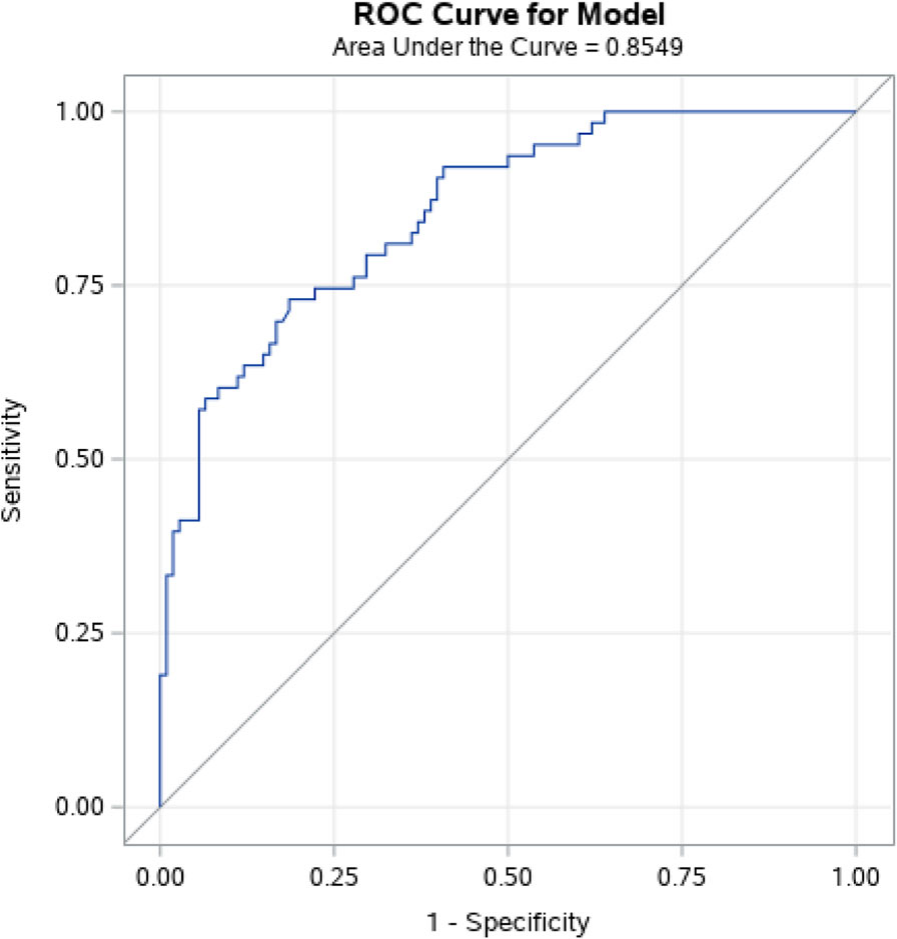
Area Under the Curve for the Multiple Logistic Regression Model

Being tenured had a strong correlation with provident fund (0.70) and grade (0.54). In Nepalese context, whether it is a public or private institution, a permanent employment (tenured status) usually requires the employer to provide both provident fund and grade (0.59) but employers can extend these benefits to non-permanent employees at any time. Since majority of the faculty worked in private sector colleges, an employer matched provident fund may have greater value towards their saving for the future. Therefore, only provident fund benefit was chosen to enter the model. Similarly, strong association was detected between current position and level of primary teaching responsibility (0.57) and total compensation above median (0.52). However, effect size between master’s program and total compensation was much lower (0.37) and both primary responsible program and total compensation were entered in the model without current position. Adequate textbook and reference book were also strongly related (0.64) and only adequate reference book which had higher correlation with outcome was selected (Fig. 2).

Table 4 shows the results from multiple logistic regression model where the job satisfaction is the function of many variables. The area under curve of the receiver operating curve showed very strong discriminatory power (AUC = 0.855) of the model. This means that the logistic regression model can accurately classify the predicted outcome as satisfied or not-satisfied for every possible pair of observations for 85.5% of the pairs. The likelihood ratio test (*p* < 0.0001) and Hosmer and Lemeshow goodness of fit statistics (*p* = 0.609) also indicate that the model is well behaved. However, a majority of the individual variables included in the model do not have a statistically significant coefficient and have a very wide odds ratio interval.

**Table 4:** Results of Multiple Logistic Regression Model

Parameter	Estimate	Pr > ChiSq	Odds Ratio	95% CI Odds Ratio	
Intercept	−7.437	0.000	Point	LL	UL
Age Group (ref: <=35 years)					
Age 36–45 years	−0.067	0.900	0.935	0.325	2.645
Age 46 and older	0.539	0.485	1.714	0.368	7.864
Male Gender	0.255	0.858	1.290	0.097	37.071
Specialty (ref: Adult Health)					
Child Health	0.563	0.347	1.756	0.543	5.785
Community Health	−0.327	0.746	0.721	0.094	5.254
Mental Health	0.533	0.517	1.704	0.330	8.571
Maternal Health	0.685	0.209	1.984	0.685	5.895
Responsibility in MSN	−0.115	0.885	0.892	0.181	4.165
Public Institution	0.085	0.866	1.088	0.403	2.910
Provident Fund Benefits	0.842	0.155	2.320	0.737	7.685
Healthcare Benefits	0.299	0.618	1.348	0.416	4.491
Decision Making (ref: Never or Rarely)					
Sometimes	1.282	0.044	3.602	1.073	13.335
Often or Always	1.575	0.019	4.833	1.353	19.111
No clinical in PM	1.194	0.069	3.299	0.924	12.357
AM Clinical after 7 AM	0.435	0.352	1.544	0.617	3.894
Weekly Work Overload (ref: < 42 h/wk)	0.683	0.198	1.979	0.720	5.844
Lower Course Load (ref: > = 125 h/yr)	0.881	0.082	2.414	0.907	6.726
Professional Development (ref: < 5 days)	0.852	0.072	2.343	0.933	6.053
Adequate Reference Books	1.072	0.050	2.922	1.026	8.953
Adequate Medical/Nursing Journals	0.842	0.090	2.321	0.888	6.299
Adequate Internet Access	1.367	0.100	3.922	0.856	23.701
Privilege to Set Questions for Test	0.495	0.378	1.641	0.541	4.966
Total compensation above median	0.093	0.852	1.098	0.408	2.940
Long Institutional Tenure (ref: < 7 yrs)	0.427	0.442	1.533	0.516	4.628
Model Performance Measures:					
Max-rescaled R-Square		0.469			
Likelihood Ratio Test (p-value)		<.0001			
Area Under Receiver Operating Curve		0.855			
Hosmer-Lameshow Goodness-of-Fit (p-value)		0.609			

Health Care benefits: Employee welfare benefit provided by an employer that provide medical care for the employee and their dependents (Father and mother or father-in-law and mother-in-law, husband/wife, children)

Table 4 showed that faculties involvement in decision making (higher job satisfaction with higher involvement levels) and adequate reference books came out to be the only strongly significant variables (*p* < 0.05) in the model. Faculties who were involved in departmental decision-making processes ‘sometimes’ and ‘often or always’ were 3.6 and 4.83 times more likely to be satisfied respectively than those who were never or rarely involved. It is interesting to note that the lowest job satisfaction (Table 1) was in the domain “operating conditions”. It suggests that job satisfaction is high when faculties are included in the decision-making process and they feel part of the ‘system’. Similarly, their job satisfaction is higher (OR = 2.92, *p* = 0.050) when reference books were adequately available for themselves and the student.

Although the adjusted odds ratios were not significant at 5% level of significance and 95% confidence interval, there were positive indicators of job satisfaction with various factors identified during the descriptive analysis. The nursing faculties were more likely to be satisfied with availability of medical or nursing journals (OR = 2.32, *p* = 0.090) and internet access (OR = 3.92, *p* = 0.100). They were 64% more likely to be satisfied (OR = 1.64, *p* = 0.378) when they were involved in setting questions for the final exams, which is often regarded a greater respect to the faculty in Nepal. They were more likely to be satisfied with their job when they did not have evening clinical (OR = 3.30, *p* = 0.069), could start their AM clinical at 8 am or later instead of earlier in the morning (OR = 1.54, *p* = 0.352), had a weekly work load of less than 42 h (OR = 1.98, *p* = 0.198), had an annual teaching load less than 125 h (OR = 2.41, *p* = 0.082), and were provided with more than 5 days of professional development opportunities (OR = 2.34, *p* = 0.072).

## Discussion

This study was conducted to explore the factors associated with job satisfaction among the nursing faculties teaching at the baccalaureate level and above in different nursing colleges under seven Universities of Nepal. The findings would help identify factors that may be modified to improve job satisfaction among these faculties, leading to higher retention of faculties and improving the overall quality of nursing education.

There have been few small-scale job satisfaction studies in Nepal with varying results prior to this study. The findings of this study support the findings of a study conducted in the Chitwan district of Nepal which shows majority of the faculties had ambivalent feeling towards their job [8]. The finding of this study also support a study conducted in the Kathmandu valley of Nepal which showed just one-third of faculty were satisfied with their job [9]. This study found that organizational commitment was an important factor in maintaining job satisfaction among nursing faculties [9].

Compared to several studies conducted in the United States of America and Australia where job satisfaction among nursing faculties was reported between 18 and 45% [10, 12–14], the 36.8% job satisfaction rate of this study seems reasonable.

Moody et al. found a significant positive relationship between years at the current institution and satisfaction with pay, coworkers, and the job in general [10]. In current study, faculties seemed to be least satisfied with their current opportunities for promotion, contingency rewards, operating conditions, payment and fringe benefits. Nursing faculties seemed to be dissatisfied with their professional promotion in this study, which is consistent with studies done in Canada [15] and the USA [16]. Promotion of nursing faculties often tied with their participation in research activities. Fourty five percentage of the nursing faculties were already working 42 h or more per week (Table 1). A promotion criterion based on number of research publications in peer reviewed journals often created additional burden on faculty. Barret et al. had found that the nursing faculties were least satisfied with an extrinsic factor like their participation in research related activities [16].

As seen in the domain wise job satisfaction scores, faculties were least satisfied with the existing operating conditions that involved excessive workloads, lots of paperwork, non-participatory decision making, and many rules and regulations that make their job more difficult. These findings are in congruence to the study by Barrett et al. which showed that the faculties were least satisfied with the excessive workload [15]. In a study conducted in the United Kingdom, McHale found that the nursing teachers were dissatisfied with excessive paperwork and suggested a reexamination of teacher’s workload to alleviate both quantitative and qualitative overload [17]. Shortage of nursing faculties, often resulting in higher student-faculty ratio, and vague job descriptions could contribute to the burden nursing faculty feel at work.

Low satisfaction regarding payment/salaries in this study was similar to the results of a study by Thies et al. [18]. The nursing faculties in this current study were satisfied with their coworkers, nature of work, communication and supervision, which supports reports from several other studies [10, 16–19].

Multiple regression analysis showed that an easy access to adequate reference books, internet, and clinical/nursing journals in their workplace were some of the key factors in the faculties’ job satisfaction. The availability of adequate references books are a cornerstone in the improvement of the knowledge and skills of both the nurses working in hospitals [20, 21] and nursing faculties working in colleges.

The faculties’ involvement in decision making process was another significant key factor of job satisfaction. A prior study conducted in the United States also showed faculties participation in decision making, an autonomy or intrinsic factor, as the key factor for job satisfaction [16].

Although we selected variables based on their high correlation with the outcome from the univariate analysis, most variables were not statistically significant in the multiple regression model. This suggests that job satisfaction outcome is the result of many interrelated variables. While individual variables were strongly related with the outcome during univariate analysis, their effect was neutralized in the presence of other variables. Collectively, the set of variables used in the model was able to discriminate job satisfaction outcome with very high degree of accuracy. The researchers suggest that the association between individual variable and outcome be taken as indicative rather than deterministic of overall job satisfaction.

### Limitations

One of the limitations of this study was that only highly motivated faculties were willing to participate in the study which may have introduced some outcome bias. Moreover, few of the participants did not have easy access to internet and did not respond to emails frequently, which limited the timely delivery of the response. Due to the lack of national registry system with the contact details of the nursing faculties working in different organization in the country, it was difficult to retrieve the contact details of the participants and some of the surveys were returned as undeliverable.

## Conclusion

Findings from this study shows only a few faculties were satisfied with their opportunities and criteria used for promotion, contingency rewards, operating conditions, payment and fringe benefits. The operating conditions included many rules and procedures resulting in lower work efficiency, and new ideas that encourage faculty innovation were not adequately promoted. Although payment was one of the lowest ranked job satisfaction domains, many non-monetary factors played key role in overall job satisfaction. While many variables had individually strong correlation with job satisfaction, the contribution from each of those variables were smaller when controlling for each other in a multivariate analysis. The researchers suggest that the association between individual variable and overall job satisfaction be taken as indicative than deterministic.

## Data Availability

Data will not be shared to ensure patient confidentiality.

## Abbreviations

BPKIHS: BP Koirala Institute of Health Sciences
KU: Kathmandu University
MN: Masters’ in Nursing
MSc Nursing: Masters’ in Science in Nursing
NAMS: National Academy of Medical Sciences
NHRC: Nepal Health Research Council
NMC: Nepal Medical College
PAHS: Patan Academy of Health Sciences
PD: Professional Development
PhD: Doctorate in philosophy
PokhU: Pokhara University
PU: Purbanchal University
SPSS: Statistical Package for the Social Sciences
TU: Tribhuvan University
